# A Comparative Analysis of Coronavirus Nucleocapsid (N) Proteins Reveals the SADS-CoV N Protein Antagonizes IFN-β Production by Inducing Ubiquitination of RIG-I

**DOI:** 10.3389/fimmu.2021.688758

**Published:** 2021-06-16

**Authors:** Yan Liu, Qi-Zhang Liang, Wan Lu, Yong-Le Yang, Ruiai Chen, Yao-Wei Huang, Bin Wang

**Affiliations:** ^1^ Department of Veterinary Medicine, Institute of Preventive Veterinary Medicine and Key Laboratory of Animal Virology of Ministry of Agriculture, Zhejiang University, Hangzhou, China; ^2^ Zhaoqing Branch Center of Guangdong Laboratory for Lingnan Modern Agricultural Science and Technology, Zhaoqing, China

**Keywords:** coronavirus, swine acute diarrhea syndrome coronavirus (SADS-CoV), nucleocapsid protein, interferon, retinoic acid-inducible gene I (RIG-I), ubiquitination

## Abstract

Coronaviruses (CoVs) are a known global threat, and most recently the ongoing COVID-19 pandemic has claimed more than 2 million human lives. Delays and interference with IFN responses are closely associated with the severity of disease caused by CoV infection. As the most abundant viral protein in infected cells just after the entry step, the CoV nucleocapsid (N) protein likely plays a key role in IFN interruption. We have conducted a comprehensive comparative analysis and report herein that the N proteins of representative human and animal CoVs from four different genera [swine acute diarrhea syndrome CoV (SADS-CoV), porcine epidemic diarrhea virus (PEDV), severe acute respiratory syndrome CoV (SARS-CoV), SARS-CoV-2, Middle East respiratory syndrome CoV (MERS-CoV), infectious bronchitis virus (IBV) and porcine deltacoronavirus (PDCoV)] suppress IFN responses by multiple strategies. In particular, we found that the N protein of SADS-CoV interacted with RIG-I independent of its RNA binding activity, mediating K27-, K48- and K63-linked ubiquitination of RIG-I and its subsequent proteasome-dependent degradation, thus inhibiting the host IFN response. These data provide insight into the interaction between CoVs and host, and offer new clues for the development of therapies against these important viruses.

## Introduction

Coronaviruses (CoVs) are an urgent public health threat, and the ongoing COVID-19 pandemic has already caused the deaths of more than 2 million people (1). The subfamily *Orthocoronavirinae* of the family *Coronaviridae* is currently classified into four genera, *Alphacoronavirus*, *Betacoronavirus*, *Gammacoronavirus*, and *Deltacoronavirus*. Increasing evidence supports that CoVs are prone to cross-species transmission (2–4). Therefore, a broad general knowledge of animal CoVs is critical for risk prediction and prevention of future zoonotic transmission events (5, 6). Swine acute diarrhea syndrome (SADS)-CoV in the genus *Alphacoronavirus,* also designated as swine enteric alphacoronavirus (SeACoV), is a newly discovered pathogen that induces diarrhea, especially in newborn-piglets, with mortality rates above 35% in southern China in 2017 (7–9). It is the fifth porcine CoV identified to date, following transmissible gastroenteritis virus (TGEV), porcine epidemic diarrhea virus (PEDV), porcine hemagglutinating encephalomyelitis virus (PHEV) and porcine deltacoronavirus (PDCoV) (10–13). SADS-CoV is closely related to bat CoV HKU2 strains and might have emerged either through genetic drift or recombination events between co-infecting CoVs (7, 9, 10).

SADS-CoV has the genome order typical of CoVs, consisting of seven independent ORFs that encode 16 non-structural proteins, 4 structural proteins and one accessory protein (7, 14). Among the structural proteins, the nucleocapsid (N) protein is highly expressed and has functions at multiple steps during viral infection (15). The N protein is highly conserved between different CoVs and is indispensable in the viral life cycle. It forms nucleocapsids with genomic RNA, promotes viral genome replication and subgenomic RNA transcription, and interacts with other viral proteins (such as the membrane [M] protein) to promote virion assembly (15). Additionally, an increasing number of studies suggest that the N protein is involved in viral evasion of the host innate immune response (16–22).

Innate immunity represents the first line of defense against pathogens and includes the type I interferon (IFN) signaling pathway, which plays an essential role in protection against viral infection (23). The IFN response starts with the recognition of pathogen associated molecular patterns (PAMPs) by pattern recognition receptors (PRRs). As RNA viruses, CoVs produce PAMPs including dsRNA and 5′-ppp RNA intermediates in the cytoplasm during replication, which can then be recognized by PRRs, specifically retinoic acid-inducible gene I (RIG-I)-like receptors (RLR) (24). After recognition and subsequent activation of RIG-I, an adaptor localized on the surface of mitochondria (mitochondrial antiviral signaling protein; MAVS) is activated by interaction between their caspase activation and recruitment domains (CARDs). MAVS then forms prion-like polymers and recruits TBK1 and IKKϵ (and other components) to form a complex that induces phosphorylation of IRF3, which subsequently promotes the production of type I IFN. This IFN production leads to expression of hundreds of IFN-stimulated genes (ISGs) in an autocrine and paracrine manner and keep the host cells in an antiviral state (25). Viruses, on the other hand, have evolved various strategies to antagonize and even benefit from the antiviral pathways of the host cell (26–28).

Various CoVs have been reported to inhibit host IFN responses during infection. PEDV inhibits IFN production by blockage of RIG-I mediated pathways (29, 30) *via* nsp1 (31), nsp5 (32), nsp15 (33) and N protein (16). TGEV papain-like protease 1 (PLP1) antagonizes INF-β production through its deubiquitinase activity (34). Severe acute respiratory syndrome (SARS)-CoV was shown to delay type I IFN signaling after infection of mice (35). Middle East respiratory syndrome (MERS)-CoV proteins NS4a, NS4b, PLP and M have been reported to inhibit IFN induction by double-stranded RNA (36–38). SARS-CoV-2 has also been reported to inhibit the host IFN response (39, 40), *via* several viral proteins (41). The avian CoV infectious bronchitis virus (IBV) has been reported to induce a delayed IFN response in primary renal cells (42). PDCoV suppressed RIG-I-dependent signaling pathways after infection of LLC-PK1 cells (43) and the nsp5, nsp15, NS6 and N proteins of PDCoV are responsible for IFN evasion (21, 22, 44–47). All together, these studies show that CoVs have evolved multiple strategies to circumvent the host IFN response.

To elucidate their roles in IFN suppression during infection, we analyzed the amino acid similarities between N proteins from several representative CoVs of four different genera, and compared targets of each N protein in IFN signaling. More importantly, we studied the mechanism of IFN inhibition by the SADS-CoV N protein by a comparative analysis. Our data show that the PAMP recognition step is a critical target for N protein suppression of IFN signaling. We also demonstrate that SADS-CoV N protein interacts with RIG-I and inducing its ubiquitination, which leads to its proteasome-dependent degradation and, consequently, the inhibition of the host IFN responses. Our data reveals one of the mechanisms by which SADS-CoV suppresses host innate immunity and provides novel clues for treatment and vaccine development against CoV infections.

## Materials and Methods

### Cells and Virus

HEK293T (ATCC, #CRL-3216) cells were purchased from the American Type Culture Collection (ATCC, Manassas, VA, USA) and cultured in Dulbecco’s modified Eagle’s medium (DMEM) (Gibco, Grand Island, NY, USA) supplemented with 10% fetal bovine serum (FBS), 100 units/ml penicillin and 100 μg/ml streptomycin. Cells were incubated at 37°C in 5% CO_2_. Sendai virus (SeV) was kindly gifted by Dr. Pinglong Xu (Life Sciences Institute, Zhejiang University).

### Antibodies and Reagents

A mouse monoclonal antibody (MAb) against flag-tag (#F3165) was purchased from Sigma-Aldrich (St Louis, MO, USA). Mouse MAbs against myc-tag (#2276), β-actin (#3700) and rabbit MAbs against myc-tag (#2278) p-IRF3 (#4947), IRF3 (#4302) were purchased from Cell Signal Tech (CST, Boston, MA, USA). Mouse MAbs against GAPDH (#ab8245) and TATA binding protein (TBP) (#ab51841) were purchased from Abcam (Cambridge, UK). HRP-conjugated goat-anti-mouse (#115-035-003) and goat-anti-rabbit (#111-0350003) polyclonal antibodies were purchased from Jackson ImmunoResearch (West Grove, PA, USA). Nuclear and cytoplasmic protein extraction kits (#78833) were purchased from Thermo Scientific (Waltham, MA, USA). Poly(I:C) sodium salt (#P1530) and polyethylenimine (PEI, MW ~25,000 kDa) (#408727) were purchased from Sigma-Aldrich. EDTA-free protease inhibitor cocktail (B14001) was purchased from Bimake (Houston, TX, USA). A dual luciferase reporter assay system (#E1960) was purchased from Promega (Madison, WI, USA).

### Plasmid Construction and Transfection

Sequences encoding the N proteins of PEDV (GenBank accession no. ANY27035), TGEV (NP_058428), SARS-CoV (YP_009825061), SARS-CoV-2 (YP_009724397), MERS-CoV (AGN70936), IBV (ABQ84805), PDCoV (AFD29191) and SADS-CoV (AWJ64267) were constructed within pRK5 vectors with a myc-tag at either the 5′- or 3′-terminus of the coding sequences. Sequences encoding human RIG-I were constructed in pRK5 vector with a flag-tag at the C-terminus. Vectors expressing RIG-IN (constitutively active mutant of RIG-I), MAVS, TBK1, IKKϵ, IRF3-5D (constitutively active mutant of IRF3), IFN-β-Luc (vector expressing luciferase underan INF-β promoter) and RL-TK were kindly gifted by Dr. Pinglong Xu (Life Sciences Institute of Zhejiang University). Special HA-tagged ubiquitins (K6-, K11-, K27, K29, K33, K48- and K63-specific) that only have lysine at their 6th, 11th, 27th, 29th, 33rd, 48th or 63rd residue were kindly provided by Dr. Zhaohui Qian (Institute of Pathogen Biology, Chinese Academy of Medical Sciences & Peking Union Medical College). For ectopic gene expression, cells were seeded in 6- or 12-well plates at 70% confluence, and transfection was performed with PEI. Briefly, indicated plasmids were mixed with PEI at a ratio of 1:1.5 (w/w) followed by incubation for 15 min at room temperature (RT) before addition to the cell culture medium. The plates were gently agitated and the medium were changed with fresh medium after incubation at 37°C for 4-6 h. A total of 1 μg plasmid was used per 12-well plate well, whereas 2 μg plasmid was used per well in 6-well plates. Cells were harvested after incubation at 37°C for 24 or 36 h.

### Luciferase Reporter Gene Assay

293T cells were seeded in 12-well plates at 70% confluence and then cotransfected with 500 ng of indicated N protein expression plasmid, 100 ng IFN-β-Luc and 50 ng RL-TK (Renilla luciferase positive control) per well. 24 h later, cells were lysed by 100 μl lysis buffer (25 mM Tris-HCl, 200 mM NaCl, 10 mM NaF, 1 mM Na_3_VO_4_, 25 mM β-glycerophosphate, 1% NP40 and protease inhibitor cocktail), and 10 μl lysate of each well was subjected to dual luciferase reporter gene assay following the manufacturer’s instructions.

### Western Blotting

293T cells were transfected with indicated plasmids as mentioned above and harvested after 24 h by addition of lysis buffer, and incubated on ice for 15 min. Cell lysates were centrifuged at 12,000 ×g for 15 min before the supernatants were either subjected to immunoprecipitation (IP) or denatured directly at 100°C for 10 min. Denatured cell lysates were separated by SDS-PAGE and transferred to PVDF membranes using 200 mA for 70 min. For immunoblotting, indicated primary antibodies were used to incubate the membranes for 3 h at RT or overnight at 4°C; HRP-conjugated goat anti-mouse or goat anti-rabbit IgG were used as secondary antibodies. The bands were visualized with chemiluminescent reagent (#1705061, Bio-Rad, Hercules, CA, USA) and were imaged by an ECL imaging system (LI-COR biosciences), and amounts of β-actin, GAPDH or TPB of each sample were used as controls to demonstrate equal loading of protein samples among lanes.

### Co-Immunoprecipitation (IP) Assay

293T cells were seeded in 6-well plates and transfected with indicated plasmids as mentioned above and lysed at 36 h post-transfection (hpt) in 300 μl lysis buffer per well. Lysates were centrifuged at 12,000 ×g for 15 min, and the supernatants were subjected to IP, with 40 μl reserved as a whole cell lysate (WCL) control. Briefly, 1 μl of antibody was added to 200 μl cell lysate and incubated on a shaker at 4°C for 2 h, followed by addition of dynabeads protein G (#10004D, Invitrogen, Carlsbad, CA, USA) which had been washed by lysis buffer three times, and then incubated for another 2 h at 4°C. The mixture was centrifuged at 5,000 ×g for 90 s and the beads were collected magnetically and washed in lysis buffer three times. Next, the beads were mixed with loading buffer and the mixture was denatured at 100°C for 10 min before analysis along with the WCL by western blot.

### Fractionation of Cytoplasmic and Nuclear Proteins

293T cells were seeded in 6-well plates at 70% confluence and transfected with indicated plasmids as mentioned above, and then harvested at 24 hpt. Cytoplasmic and nuclear proteins were fractionated and extracted by using a nuclear and cytoplasmic protein extraction kit (Thermo Scientific) following the manufacturer’s instructions. Extracted proteins were quantified using a BCA protein assay kit (Thermo Scientific, #23225), and an equal amount of sample was used for western blot analysis.

### Statistical Analyses

Data were analyzed by GraphPad Prism 6 software and Student’s *t*-tests were performed to determine significance. Data were expressed as mean ± standard deviation (SD) of three independent experiments. *P* values < 0.05 were considered to be statistically significant.

## Results

### Sequence Comparison of CoV N Proteins

**Figure 1:**
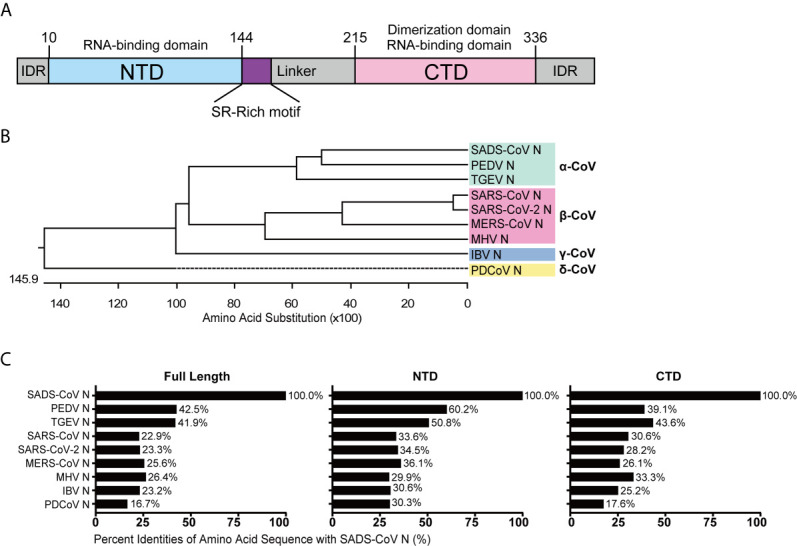
Amino acid sequence similarity between the N proteins of SADS-CoV and other members of the Coronavirinae. **(A)** Schematic representation of SADS-CoV N protein domains. Three intrinsically disordered regions (IDR), the N-terminal domain (NTD) and C-terminal domain (CTD) are shown. The charged Ser/Arg (SR)-rich motif (coloured purple) is shown. **(B)** Phylogenetic analysis of N proteins of representative coronaviruses from each genus. **(C)** The alignment was conducted by clustalW X, and the figure was generated by GraphPad Prism 7.0 according to the similarity calculated by DNASTAR MegAlign.

To analyze their conservation, the SADS-CoV N protein was compared with those of other CoVs using ClustalW, and amino acid sequence similarities were calculated. The full length N protein of SADS-CoV contains 375 amino acids, with the NTD and CTD domains predicted based on sequence alignment with HCoV-NL63 N protein from the genus *Alphacoronavirus*, which is the only N protein whose structure has been solved so far (48). The putative NTD spans from residue 10 to 144 while the CTD spans from residue 215 to 336 (**Figure 1A**), similar to other CoV N proteins in length. To illustrate the evolutionary relationship, we constructed a phylogenetic tree based on N protein amino acid sequences from representative CoVs of each genus (**Supplementary Figure S1**). As expected, the N protein amino acid sequence identity was consistent with the genus clustering observed for the whole genome (**Figure 1B**). Next, we analyzed the amino acid sequence similarities between the full-length, NTD and CTD sequences from the N proteins of various representative CoVs. As shown in **Figure 1C**, the similarity to SADS-CoV among the alphacoronaviruses was relatively high, 42.5% for PEDV and 41.9% for TGEV. The similarities of N proteins from the other genera ranged from 26.4% for mouse hepatitis virus (MHV) to 16.7% for PDCoV. Notably, there was greater similarity in the NTD and CTD than in the full sequence, suggesting that the intrinsically disordered regions (IDRs) are more divergent. The NTDs were more highly conserved (60.2-29.9%) than the CTDs (43.6-17.6%), although this was not statistically significant (*P*=0.1342) (**Figure 1C**). These data demonstrate that N proteins of CoVs are relative conserved, especially within genus, thus we hypothesized that they might exhibit similar functions with respect to evasion of innate immunity.

### CoV N Proteins Inhibit IFN Production by Interfering With Different Stages of the Signaling Pathway

**Figure 2:**
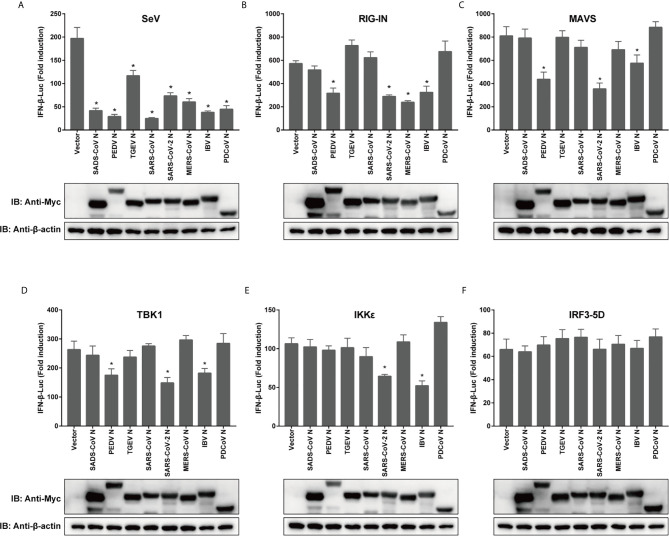
Coronaviral N proteins antagonize IFN-β promoter activation *via* the RLR signaling pathway. 293T cells were transfected with expression vectors carrying IFN-β-Luc, RL-TK, and indicated CoV N genes for 12 h prior to SeV infection **(A)**, or cotransfected along with constructs expressing RIG-IN **(B)**, MAVS **(C)**, IKKϵ **(D)**, TBK1 **(E)** or IRF3-5D **(F)**. Luciferase activity was measured at 24 h post-transfection or 12 h after SeV infection. IFN-β activity is expressed as the fold change of firefly luciferase activity normalized by renilla luciferase activity compared to the empty vector control. The expression of N protein for each experiment was determined by western blotting. All experiments were performed three times, and pairwise differences in means were analyzed with Student’s t-test; *p < 0.05.

To compare the mechanisms by which CoV N proteins from different genera inhibit IFN production, a dual luciferase reporter gene assay was performed in transfected cells, using a RIG-I activating model virus, SeV, to activate RLR signaling. To ensure that the effects of each N protein from distinct CoVs were comparable, 293T cells were used in all reporter assays. The activity of the IFN-β promoter was inhibited by all the N proteins we tested, including N proteins of alphacoronaviruses TGEV, PEDV, SADS-CoV, betacoronaviruses SARS-CoV, SARS-CoV-2, MERS-CoV, the gammacoronavirus IBV and the deltacoronavirus PDCoV (**Figure 2A**). Next, we confirmed the effects of N protein expression on IFN-β promoter activity, this time induced by RIG-IN, a truncated, constitutively active form of RIG-I (49). The N proteins from PEDV, SARS-CoV-2, MERS-CoV and IBV were able to inhibit IFN-β promoter activity in these tests, suggesting that they target downstream of RIG-I or interfere with the interaction between RIG-I and MAVS (**Figure 2B**).

Moving downstream, we wanted to test the effect of overexpression of other key signaling components on the observed inhibition by CoV N proteins. For the mitochondrial adapter MAVS, the N protein of TGEV, SADS-CoV, SARS-CoV, MERS-CoV, or PDCoV lost its inhibitive effect (**Figure 2C**). Notably, the N proteins of PEDV, SARS-CoV-2 and IBV were all capable of inhibiting the activation of IFN-β pathway by TBK1, which is recruited to form complexes with MAVS that phosphorylate and subsequently activate IRF3. The PEDV N protein did not block IKKϵ induction of IFN-β, although the N proteins of SARS-CoV-2 and IBV did (**Figures 2D, E**). However, all N proteins in this study lost their inhibitory effect when the pathway was activated by IRF3-5D (**Figure 2F**), which is a constitutively active form of IRF3 that is able to enter the nucleus to spontaneously activate IFN. To confirm the expression of N proteins, western blotting was conducted and the expression levels of N proteins have been shown (**Figure 2**). These data indicate that N proteins of different CoVs use various strategies to inhibit IFN expression, and even N proteins with relatively high similarity might inhibit IFN expression by targeting different stages of the signaling cascade.

### The N Protein of SADS-CoV Inhibits IRF3 Phosphorylation and Nuclear Translocation Induced by SeV, But Not by RIG-IN

**Figure 3:**
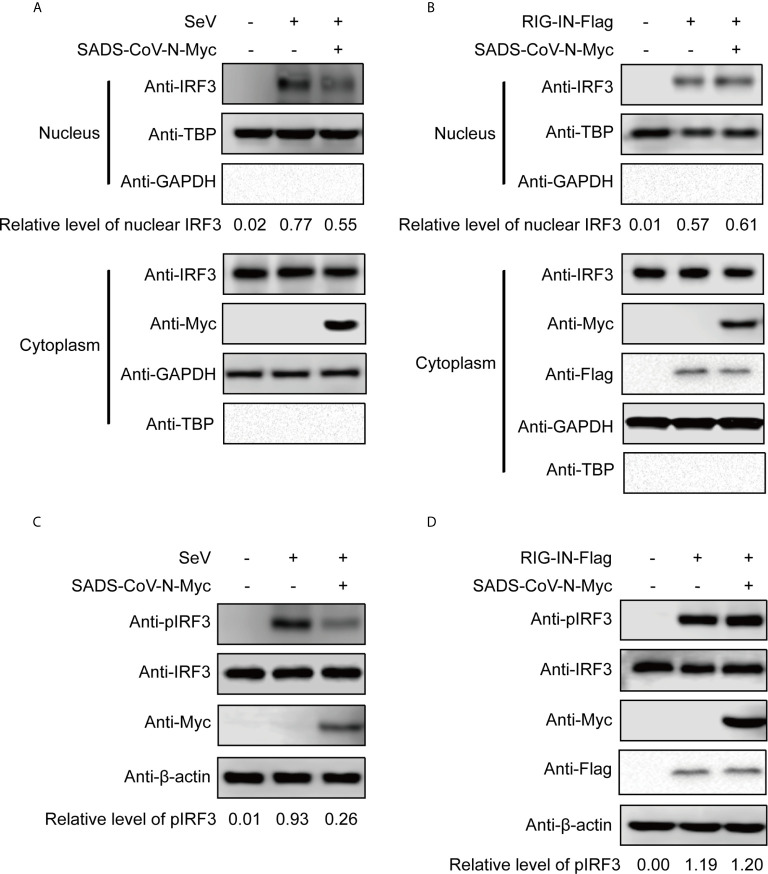
The SADS-CoV N protein inhibits the phosphorylation and nuclear translocation of IRF3 induced by SeV. 293T cells were transfected with an expression vector carrying the SeVCoV N gene, and IRF3 was activated either by SeV infection **(A, C)** or RIG-IN cotransfection **(B, D)**. Endogenous IRF3 nuclear translocation **(A, B)** and phosphorylation **(C, D)** were detected by western blot at 36 h post-transfection in nuclear and cytoplasmic fractions. GAPDH, TBP and β-actin were used as loading controls in cytoplasm, nucleus and whole cell lysates, respectively. Quantification of each band intensity was done using Image Lab software 6.0.

We wanted to explore in greater detail the mechanism of IFN inhibition utilized by the recently emerging SADS-CoV. Since the previous experiment suggested that the SADS-CoV N protein inhibits IFN-β promoter after SeV infection but not after induction by RIG-IN (**Figures 2A, B**), we took a closer look at the effect of SADS-CoV N protein on IRF3 activity. Activation of IRF3 *via* phosphorylation and nuclear translocation is essential for its induction of IFN-β production (50). IRF3 nuclear translocation was observed in cellular fractionation experiments, detecting IRF3 phosphorylation by western blot with S396 phosphorylated IRF3 MAb. As shown in **Figure 3**, IRF3 nuclear translocation and phosphorylation were effectively induced by both SeV infection and RIG-IN overexpression. Notably, the observed IRF3 nuclear translocation and phosphorylation induced by SeV infection were inhibited by the SADS-CoV N protein (**Figures 3A, C**). However, the SADS-CoV N protein had no significant effect on IRF3 induced by RIG-IN (**Figures 3B**), confirming the results of the previous dual-luciferase reporter experiments and suggesting that SADS-CoV N blocks IFN expression by targeting RIG-I or the process upstream of RIG-I.

### The N Protein of SADS-CoV Interacts With RIG-I Independently of Its RNA Binding Activity

**Figure 4:**
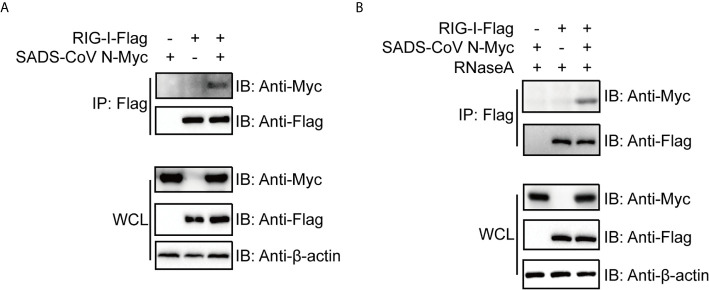
The SADS-CoV N protein binds to RIG-I in a manner independent of RNA. Myc-tagged SADS-CoV N protein and flag-tagged full length RIG-I were coexpressed in 293T cells. At 36 h post-transfection, cell lysates were either immunoprecipitated (IP) by antibodies against flag-tag directly **(A)** or treated with 100 μg/ml RNase A on ice for 1 h before IP **(B)**. SADS-CoV N and RIG-I in IP products and whole cell lysates were detected with western blot using antibodies against myc-tag and flag-tag, respectively.

The above results led us to speculate that the SADS-CoV N protein inhibits IFN signaling by interfering with the function of RIG-I. As a PRR for RNA PAMPs in the cytoplasm, RIG-I binds directly to RNA and subsequently activates downstream signaling (51). To investigate the mechanism by which SADS-CoV N inhibits RIG-I, we first asked whether there was a direct protein-protein interaction between them. We cotransfected 293T cells with flag-tagged full-length RIG-I and myc-tagged SADS-CoV-N, followed by co-IP at 36 hpt by using anti-flag-tag antibodies. As shown in **Figure 4A**, SADS-CoV N protein was detected in the IP product, suggesting direct interaction between SADS-CoV N protein and RIG-I. Because N protein has RNA binding activity similar to RIG-I, we investigated whether the interaction is dependent on RNA binding of these two proteins. For this aim, the cell lysates were treated with RNaseA before co-IP was performed. As shown in **Figure 4B**, the SADS-CoV N protein was detected in the IP product even after RNaseA treatment, demonstrating the interaction with RIG-I was independent of shared RNA binding.

### The SADS-CoV N Protein Induces Ubiquitination of RIG-I Leading to Its Degradation

**Figure 5:**
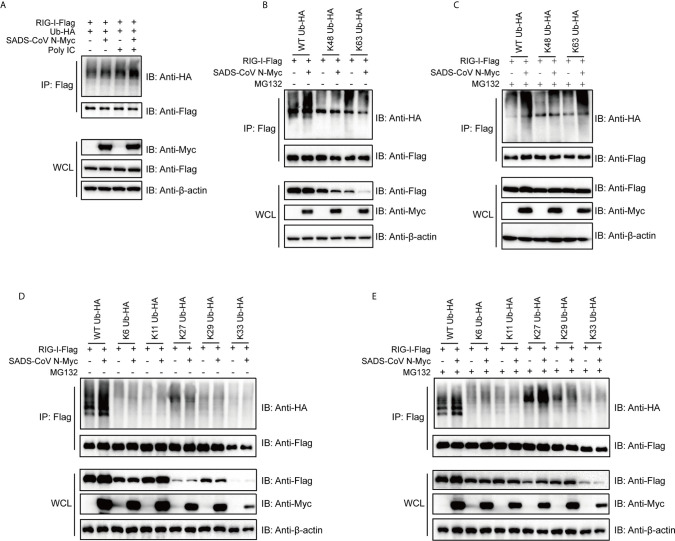
The SADS-CoV N protein induces ubiquitination of RIG-I and mediates proteasomal degradation of polyubiquitin-linked RIG-I. **(A)** HA-tagged wild type ubiquitin, flag-tagged full-length RIG-I and empty vector or SADS-CoV N were coexpressed in 293T cells. RIG-I signaling was activated by transfection with poly(I:C), and ubiquitination of RIG-I was measured by detection of HA-tags in immunoprecipitation products using anti-flag antibodies at 36 h post-transfection (hpt). **(B, C)** Flag-tagged full length RIG-I and empty vector or SADS-CoV N protein were coexpressed in 293T cells, along with HA-tagged wild type, K63- or K48-ubiquitin with **(B)** or without **(C)** 10 μM of MG132 treatment at 4 hpi until measurement of RIG-I ubiquitination at 36 hpt. **(D, E)** Experiment was repeated as in **(B, C)**, except that K6-, K11-, K27-, K29-, K33-ubiquitin were used.

The ubiquitination of RIG-I has been proven to be essential for activation of the RIG-I signaling pathway (52–54). Previous studies have identified several ubiquitination states of RIG-I that regulate its activity in different ways (52, 55, 56). To investigate the mechanism by which SADS-CoV impacts RIG-I, we determined whether the SADS-CoV N protein regulates ubiquitination of RIG-I. 293T cells were cotransfected with ubiquitin, RIG-I, and SADS-CoV N or empty vector, with or without poly(I:C) (which is a specific agonist for RLR). Cells were harvested at 36 hpt and the level of ubiquitination of RIG-I was analyzed by using co-IP and western blot. Poly(I:C) significantly induced ubiquitination of RIG-I (**Figure 5A**, Lane 3), and, interestingly, SADS-CoV N enhanced the ubiquitination of RIG-I both in the presence and absence of poly(I:C) (**Figure 5A**, Lanes 2 and 4). To determine which type of ubiquitination was upregulated by the SADS-CoV N protein, K48- and K63-ubiquitin were used. Whereas wild-type ubiquitination of RIG-I was significantly enhanced by the SADS-CoV N protein (**Figure 5A**), K63-ubiquitination was decreased while no significant change in K48-ubiquitination was found (**Figure  5B**). Most interestingly, we found significant decreases in the amount of RIG-I either in the presence of K48-ubiquitin or K63-ubiquitin in whole cell lysates, but not the wild-type ubiquitin, and this decreased amount was further reduced by SADS-CoV N coexpression. Since proteasome-dependent degradation is a common outcome of ubiquitination modification (57), we asked whether the reduced RIG-I levels in the whole cell lysate was caused by proteasome-mediated protein degradation. MG132 treatment was performed at 4 hpt to inhibit proteasome activity. The RIG-I level in whole cell lysates completely recovered after MG132 treatment, both in the presence of K48-ubiquitin and K63-ubiquitin (**Figure 5C**). In addition, both K48- and K63-ubiquitin modification of RIG-I were enhanced by the SADS-CoV N protein after MG132 treatment, indicating that SADS-CoV N induces ubiquitination of RIG-I leading to its proteasome-dependent degradation.

To further explore which type of RIG-I ubiquitination was being induced by SADS-CoV N, K6-, K11-, K27-, K29- and K33-ubiquitin, covering all the lysines present in the amino acid sequence of ubiquitin, were used to perform co-IP assay. Interestingly, K27-ubiquination was significantly increased by SADS-CoV N when cells were treated with MG132, whereas only a slight change in K27-ubiquitination was induced without MG132 treatment (**Figures 5D, E**, Lanes 7 and 8). Notably, in K27-ubiquitin co-transfected cells, SADS-CoV N increased the level of RIG-I in whole cell lysate in the presence of MG132, indicating a proteasome-dependent degradation of RIG-I mediated by K27-ubiquitination (**Figure 5E**).

## Discussion

Viruses have been reported to interfere with innate immunity and delay IFN responses, an ability that creates a suitable replication environment typical for infections by several CoVs (29, 41, 43, 58–63). SADS-CoV is a newly emerged porcine CoV with potential for zoonotic transmission (7), and it has been proven to antagonize IFN-β production (58). However, the mechanism used by the SADS-CoV N protein in interfering with host innate immunity remains to be clarified.

The N protein is more highly conserved than other CoV structural proteins such as the spike protein, which may be due to its vital functions and the lower selective pressure placed on it. Thus, we wanted to see whether N protein amino acid similarity was predictive of a functional role in suppression of IFN response (**Supplementary Figure S1**). For the various N proteins tested, ability to suppress IFN response was not clearly related to the amino acid sequence similarity (**Figures 1** and **2**). SARS-CoV-2 N inhibited IFN promoter activity induced by RIG-IN, MAVS, TBK1 and IKKϵ, whereas the SARS-CoV N protein did not (**Figures 2B–D**), despite an overall amino acid similarity of 91.2%, the highest among N proteins tested in this study (data not shown). This shows the importance of tertiary structure in determining protein function, which is not always evident from the simple amino acid sequence. To date, there is no available complete tertiary structure of the CoV N protein, whereas structures of the NTD and CTD have been reported for various CoV N proteins (48, 64–68). As described by earlier reports, the core NTD and CTD structures of CoV N proteins are highly conserved (48, 64–68); however, little evidence has been found so far to link the CoV N protein antagonism of host innate immunity to the tertiary structure of N protein, which is urgently needed to demonstrate the structural basis of the innate immune antagonistic activities of N proteins.

**Figure 6:**
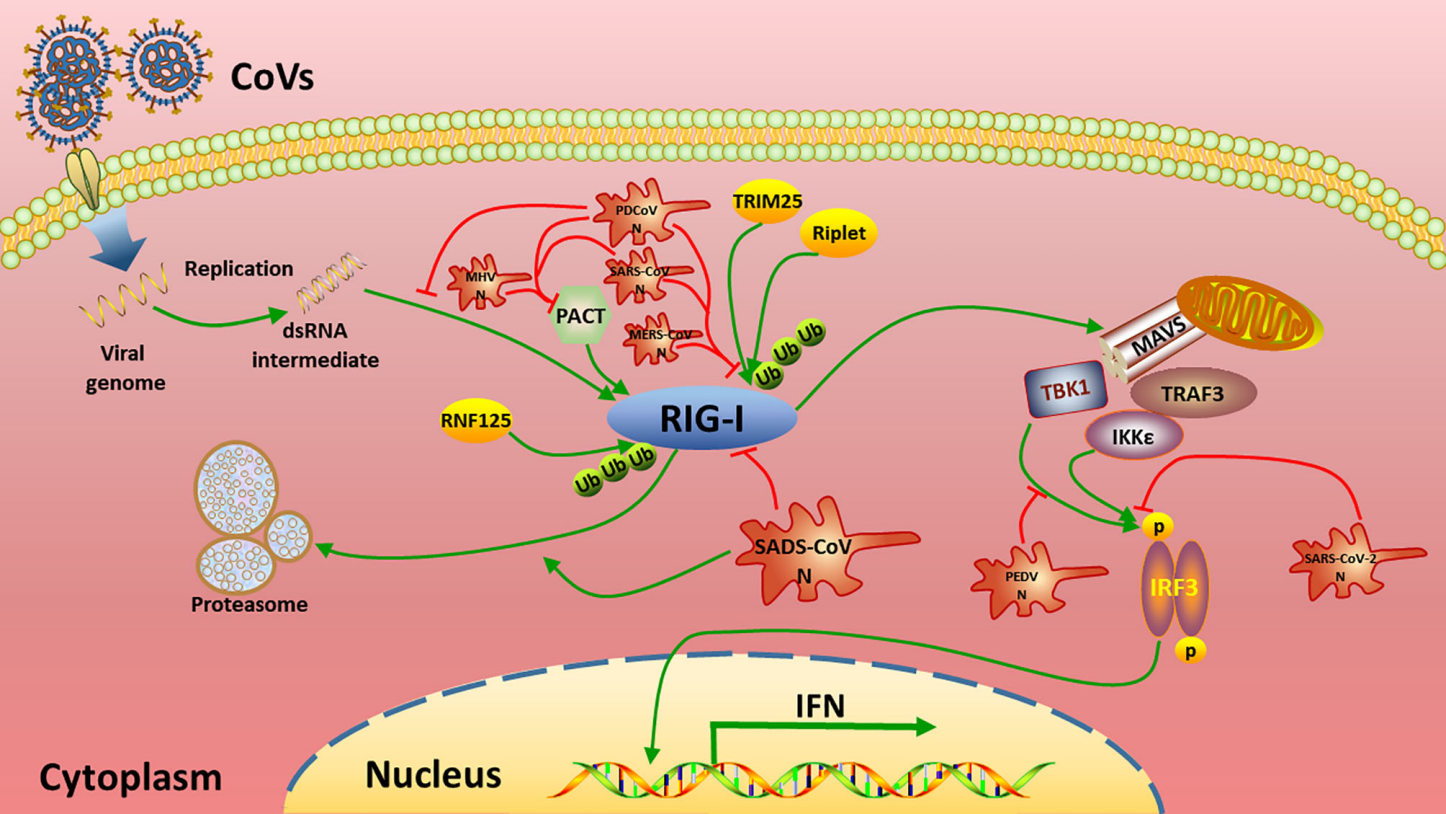
Summary of current mechanisms underlying the inhibition of interferon (IFN) signaling by distinct CoV N proteins. The N proteins of SADS-CoV, MHV, SARS-CoV, MERS-CoV, PDCoV target the very beginning step of RLR signaling pathway (RIG-I) to inhibit IFN production, while PEDV N and SARS-CoV-2 N target TBK1 and/or downstream of TBK1 to circumvent IFN production.

To date, N proteins from several CoVs have been proven to inhibit host IFN response (**Figure 6**). The PEDV N protein has been reported to antagonize host IFN responses by interacting with TBK1 directly (16). The SARS-CoV N interacts with TRIM25 and protein activator of protein kinase R (PACT) directly to interfere with activation of RIG-I (17, 18). The MERS-CoV N suppresses type I and type III IFN induction by interaction with TRIM25 (18, 69). The MHV N protein interacts with PACT to inhibit activation of RIG-I (17). The PDCoV N protein interacts with RIG-I directly and interferes with binding of dsRNA and PACT to RIG-I (21, 22). The N protein of SARS-CoV-2, the etiological agent of COVID-19, also has been shown to inhibit IFN induced by SeV, poly(I:C), RIG-I, MAVS, TBK1, and IKKϵ (70). Our current results are consistent with these previous studies (**Figures 2** and **6**), but go further in suggesting that SADS-CoV N targets the very early steps of the IFN response, and may directly interfere with activation of RIG-I (**Figures 2A, B** and **3**). During preparation of our manuscript, Zhou et al. reported that SADS-CoV N blocked interaction between TBK1 and TRAF3 (71). CoV N proteins may target multiple host protein simultaneously such as SARS-CoV N protein interacting with TRIM25 and PACT (17, 18). Since SADS-CoV infection interfere with host IFN signaling upstream of MAVS (58), and since SADS-CoV N appeared not to suppress IFN-b induced by TBK1, IKKe and IRF3-5D, which is indicated by the comparative analysis in **Figures 2D–F**, we speculate that interference *via* RIG-I is likely a dominant mode for SADS-CoV N protein to inhibit RLR signaling (**Figure 6**).

RLRs are cytoplasmic PRRs that recognize RNA PAMPs, and they have been proven to play critical roles in IFN induction during CoV infection (24, 72, 73). There are several CoV N proteins that have been shown to suppress host IFN response by targeting RIG-I or related proteins, such as those of SARS-CoV, MERS-CoV, MHV and PDCoV (18, 20–22, 69). These studies led us to look closely at RIG-I as a likely target for CoV to circumvent the host IFN response. Consistently, our data demonstrated that the SADS-CoV N protein targets RIG-I to inhibit IFN-β promoter activity (**Figure 2**). Our co-IP results indicate that SADS-CoV N protein interacted with RIG-I independently of RNA binding activity (**Figure 4**), making it the only alphacoronavirus N protein that interacts with RIG-I directly. Recently, PDCoV, a deltacoronavirus that infects swine and has the potential for interspecies transmission, was shown to bind RIG-I with its N protein (21, 22).

Full activation of RIG-I depends on multiple post-translational modifications beyond the simple presence of PAMPs. It is reported that ubiquitination, removal of inhibitory phosphorylation as well as some cofactors, such as PACT, are essential for full activation of RIG-I (52, 74, 75). Among these, ubiquitination has been reported to be critical for regulation of RIG-I, allowing versatile outcomes in IFN signaling depending on the type of ubiquitin modification (52, 55, 56, 76, 77). One common outcome of ubiquitination is proteasome-dependent degradation of the target protein. To date, many viruses have been demonstrated to modulate ubiquitination of RIG-I in order to interfere with host immune signaling (78, 79). For instance, the E6 oncoprotein of human papillomavirus (HPV) interacts with TRIM25, which is a ubiquitin E3 ligase that triggers K63-linked ubiquitination of RIG-I, thereby inhibiting the activation of downstream signaling (80). Paramyxovirus V proteins interact with RIG-I as well as TRIM25 to antagonize RIG-I-mediated IFN induction (81). Our data demonstrated that the SADS-CoV N protein enhances K27-, K48- and K63-linked ubiquitination of RIG-I (**Figure 5**). Previous studies reported that K48-linked ubiquitination of RIG-I leads to degradation while K63-linked ubiquitination leads to activation of RIG-I (53, 77). Consistently, K48-linked ubiquitination of RIG-I induced by SADS-CoV N protein led to proteasome-dependent degradation of RIG-I in our study (**Figures 5B, C**). Unexpectedly, SADS-CoV N-mediated K63-ubiquitination of RIG-I also led to degradation of RIG-I (**Figures 5B** **, C**). There are multiple E3 ligases that mediate K63-linked ubiquitination of RIG-I identified so far, such as TRIM25, Riplet, MEX3C and TRIM4 (53). These E3 ligases deliver a K63-linked polyubiquitin moiety to various domains of RIG-I, and might lead to divergent physiological outcome. The role of K27-linked ubiquitination of RIG-I is still elusive. Some components of the RLR signaling pathway such as MAVS (82) and IRF3 (83) have been reported to be modified by K27-linked polyubiquitination leading to autophagic degradation, suggesting that this type of ubiquitination plays an important role in host antiviral innate immunity. The K27-linked ubiquitination of RIG-I mediated by SADS-CoV N protein did not decrease the level of RIG-I in whole cell lysates (**Figure 5D**, lanes 7 and 8), indicating a mode of action other than degradation by this type of modification on RIG-I activation. More importantly, while the wild type ubiquitination induced by SADS-CoV N protein did not lead to significant reduction of amount of RIG-I in whole cell lysates (**Figure 5**), we speculate that K48- and K63-linked ubiquitination might not be the major form of ubiquitination induced by SADS-CoV N protein.

In conclusion, the present study provides evidence, by a comprehensive comparative analysis with representative CoVs from different genera, which the N protein of SADS-CoV interacts with RIG-I independent of its RNA binding activity. The N protein also induces ubiquitination and subsequent proteasome-dependent degradation of RIG-I. These effects may contribute to the ability of SADS-CoV N to inhibit IFN. Our data provide insight into interaction between SADS-CoV and host, and offer new clues for therapeutic treatment and vaccine development against SADS-CoV.

## Data Availability Statement

The raw data supporting the conclusions of this article will be made available by the authors, without undue reservation.

## Author Contributions

Y-WH and BW conceived and designed the study and critically revised the manuscript. YL and Q-ZL performed the experiments and conducted data analysis. WL, Y-LY, and RC helped in experimental implementation. YL and Y-WH wrote the manuscript. All authors contributed to the article and approved the submitted version.

## Funding

This work was supported by the National Natural Science Foundation of China (32041003 and 31872488) and the Zhaoqing Branch Center of Guangdong Laboratory for Lingnan Modern Agricultural Science and Technology.

## Conflict of Interest

The authors declare that the research was conducted in the absence of any commercial or financial relationships that could be construed as a potential conflict of interest.

## Supplementary Material

Supplementary Figure S1 Multiple amino acid sequence alignment of the N proteins among the representative CoVs shown in Figure 1B. The alignment was conducted by ClustalW and the image was modified by Jalview 2.2. The default color scheme was used (http://www.jalview.org/help/html/colourSchemes/clustal.html) and 50% conservation was set to be the threshold for color visibility. The sequences corresponding to the NTD, CTD and RS-rich region of the SADS-CoV N were marked above.

## References

[1] Zhou P Yang XL Wang XG Hu B Zhang L Zhang W. A Pneumonia Outbreak Associated With a New Coronavirus of Probable Bat Origin. Nature (2020) 579(7798):270–3. 10.1038/s41586-020-2012-7 PMC7095418 32015507

[2] Rodrigues J Barrera-Vilarmau S Mc Teixeira J Sorokina M Seckel E Kastritis PL. Insights on Cross-Species Transmission of SARS-CoV-2 From Structural Modeling. PLoS Comput Biol (2020) 16(12):e1008449. 10.1371/journal.pcbi.1008449 33270653 PMC7714162

[3] Cui J Li F Shi ZL. Origin and Evolution of Pathogenic Coronaviruses. Nat Rev Microbiol (2019) 17(3):181–92. 10.1038/s41579-018-0118-9 PMC7097006 30531947

[4] Latinne A Hu B Olival KJ Zhu G Zhang L Li H. Origin and Cross-Species Transmission of Bat Coronaviruses in China. Nat Commun (2020) 11(1):4235. 10.1038/s41467-020-17687-3 32843626 PMC7447761

[5] Schlottau K Rissmann M Graaf A Schön J Sehl J Wylezich C. Sars-CoV-2 in Fruit Bats, Ferrets, Pigs, and Chickens: An Experimental Transmission Study. Lancet Microbe (2020) 1(5):e218–e25. 10.1016/s2666-5247(20)30089-6 PMC7340389 32838346

[6] Yang YL Qin P Wang B Liu Y Xu GH Peng L. Broad Cross-Species Infection of Cultured Cells by Bat Hku2-Related Swine Acute Diarrhea Syndrome Coronavirus and Identification of Its Replication in Murine Dendritic Cells *In Vivo* Highlight Its Potential for Diverse Interspecies Transmission. J Virol (2019) 93(24):e01448–19. 10.1128/jvi.01448-19 PMC6880172 31554686

[7] Pan Y Tian X Qin P Wang B Zhao P Yang YL. Discovery of a Novel Swine Enteric Alphacoronavirus (SeACoV) in Southern China. Vet Microbiol (2017) 211:15–21. 10.1016/j.vetmic.2017.09.020 29102111 PMC7117260

[8] Xu Z Zhang Y Gong L Huang L Lin Y Qin J. Isolation and Characterization of a Highly Pathogenic Strain of Porcine Enteric Alphacoronavirus Causing Watery Diarrhoea and High Mortality in Newborn Piglets. Transbound Emerg Dis (2019) 66(1):119–30. 10.1111/tbed.12992 PMC7168553 30103259

[9] Zhou P Fan H Lan T Yang XL Shi WF Zhang W. Fatal Swine Acute Diarrhoea Syndrome Caused by an HKU2-Related Coronavirus of Bat Origin. Nature (2018) 556(7700):255–8. 10.1038/s41586-018-0010-9 PMC7094983 29618817

[10] Yang YL Yu JQ Huang YW. Swine Enteric Alphacoronavirus (Swine Acute Diarrhea Syndrome Coronavirus): An Update Three Years After Its Discovery. Virus Res (2020) 285:198024. 10.1016/j.virusres.2020.198024 32482591 PMC7229464

[11] Huang YW Dickerman AW Pineyro P Li L Fang L Kiehne R. Origin, Evolution, and Genotyping of Emergent Porcine Epidemic Diarrhea Virus Strains in the United States. MBio (2013) 4(5):e00737–13. 10.1128/mBio.00737-13 PMC3812708 24129257

[12] Opriessnig T Huang YW. Coronavirus Disease 2019 (COVID-19) Outbreak: Could Pigs be Vectors for Human Infections? Xenotransplantation (2020) 27:e12591. 10.1111/xen.12591 32222047 PMC7169798

[13] Wang B Liu Y Ji CM Yang YL Liang QZ Zhao P. Porcine Deltacoronavirus Engages the Transmissible Gastroenteritis Virus Functional Receptor Porcine Aminopeptidase N for Infectious Cellular Entry. J Virol (2018) 92(12):e00318–18. 10.1128/JVI.00318-18 PMC5974500 29618640

[14] Yang YL Liang QZ Xu SY Mazing E Xu GH Peng L. Characterization of a Novel Bat-HKU2-Like Swine Enteric Alphacoronavirus (SeACoV) Infection in Cultured Cells and Development of a SeACoV Infectious Clone. Virology (2019) 536:110–8. 10.1016/j.virol.2019.08.006 PMC7112019 31419711

[15] McBride R van Zyl M Fielding BC. The Coronavirus Nucleocapsid Is a Multifunctional Protein. Viruses (2014) 6(8):2991–3018. 10.3390/v6082991 25105276 PMC4147684

[16] Ding Z Fang L Jing H Zeng S Wang D Liu L. Porcine Epidemic Diarrhea Virus (PEDV) Nucleocapsid Protein Antagonizes IFN-β Production by Sequestering the Interaction Between IRF3 and TBK1. J Virol (2014) 88:8936–450. 10.1128/jvi.00700-14 24872591 PMC4136253

[17] Ding Z Fang L Yuan S Zhao L Wang X Long S. The Nucleocapsid Proteins of Mouse Hepatitis Virus and Severe Acute Respiratory Syndrome Coronavirus Share the Same IFN-Beta Antagonizing Mechanism: Attenuation of PACT-Mediated RIG-I/ MDA5 Activation. Oncotarget (2017) 8:49655–70. 10.18632/oncotarget.17912 28591694 PMC5564796

[18] Hu Y Li W Gao T Cui Y Jin Y Li P. The Severe Acute Respiratory Syndrome Coronavirus Nucleocapsid Inhibits Type I Interferon Production by Interfering With TRIM25-Mediated Rig-I Ubiquitination. J Virol (2017) 91(8):e02143–16. 10.1128/jvi.02143-16 28148787 PMC5375661

[19] Kopecky-Bromberg SA Martinez-Sobrido L Frieman M Baric RA Palese P. Severe Acute Respiratory Syndrome Coronavirus Open Reading Frame (ORF) 3b, ORF 6, and Nucleocapsid Proteins Function as Interferon Antagonists. J Virol (2007) 81(2):548–57. 10.1128/JVI.01782-06 PMC1797484 17108024

[20] Lu X Pan J Tao J Guo D. Sars-CoV Nucleocapsid Protein Antagonizes IFN-Beta Response by Targeting Initial Step of IFN-Beta Induction Pathway, and its C-terminal Region Is Critical for the Antagonism. Virus Genes (2011) 42(1):37–45. 10.1007/s11262-010-0544-x 20976535 PMC7088804

[21] Chen J Fang P Wang M Peng Q Ren J Wang D. Porcine Deltacoronavirus Nucleocapsid Protein Antagonizes IFN-β Production by Impairing dsRNA and PACT Binding to RIG-I. Virus Genes (2019) 55:520–31. 10.1007/s11262-019-01673-z 31129785 PMC7088841

[22] Likai J Shasha L Wenxian Z Jingjiao M Jianhe S Hengan W. Porcine Deltacoronavirus Nucleocapsid Protein Suppressed IFN-β Production by Interfering Porcine RIG-I dsRNA-Binding and K63-Linked Polyubiquitination. Front Immunol (2019) 10:1024. 10.3389/fimmu.2019.01024 31143181 PMC6521028

[23] Takeuchi O Akira S. Innate Immunity to Virus Infection. Immunol Rev (2009) 227(1):75–86. 10.1111/j.1600-065X.2008.00737.x 19120477 PMC5489343

[24] Takeuchi O Akira S. MDA5/RIG-I and Virus Recognition. Curr Opin Immunol (2008) 20(1):17–22. 10.1016/j.coi.2008.01.002 18272355

[25] Ramos HJ Gale M Jr. RIG-I Like Receptors and Their Signaling Crosstalk in the Regulation of Antiviral Immunity. Curr Opin Virol (2011) 1(3):167–76. 10.1016/j.coviro.2011.04.004 PMC3177754 21949557

[26] Beachboard DC Horner SM. Innate Immune Evasion Strategies of DNA and RNA Viruses. Curr Opin Microbiol (2016) 32:113–9. 10.1016/j.mib.2016.05.015 PMC4983539 27288760

[27] Garcia-Sastre A. Ten Strategies of Interferon Evasion by Viruses. Cell Host Microbe (2017) 22(2):176–84. 10.1016/j.chom.2017.07.012 PMC5576560 28799903

[28] Xu LD Zhang F Peng L Luo WT Chen C Xu P. Stable Expression of a Hepatitis E Virus (Hev) RNA Replicon in Two Mammalian Cell Lines to Assess Mechanism of Innate Immunity and Antiviral Response. Front Microbiol (2020) 11:603699. 10.3389/fmicb.2020.603699 33424806 PMC7793998

[29] Cao L Ge X Gao Y Herrler G Ren Y Ren X. Porcine Epidemic Diarrhea Virus Inhibits dsRNA-Induced Interferon-Beta Production in Porcine Intestinal Epithelial Cells by Blockade of the RIG-I-Mediated Pathway. Virol J (2015) 12:127. 10.1186/s12985-015-0345-x 26283628 PMC4539884

[30] Guo L Luo X Li R Xu Y Zhang J Ge J. Porcine Epidemic Diarrhea Virus Infection Inhibits Interferon Signaling by Targeted Degradation of STAT1. J Virol (2016) 90(18):8281–92. 10.1128/JVI.01091-16 PMC5008104 27384656

[31] Wang D Fang L Shi Y Zhang H Gao L Peng G. Porcine Epidemic Diarrhea Virus 3c-Like Protease Regulates Its Interferon Antagonism by Cleaving Nemo. J Virol (2016) 90(4):2090–101. 10.1128/JVI.02514-15 PMC4733996 26656704

[32] Zhang Q Shi K Yoo D. Suppression of Type I Interferon Production by Porcine Epidemic Diarrhea Virus and Degradation of CREB-Binding Protein by Nsp1. Virology (2016) 489:252–68. 10.1016/j.virol.2015.12.010 PMC7111358 26773386

[33] Deng X van Geelen A Buckley AC O’Brien A Pillatzki A Lager KM. Coronavirus Endoribonuclease Activity in Porcine Epidemic Diarrhea Virus Suppresses Type I and Type III Interferon Responses. J Virol (2019) 93:e02000–18. 10.1128/jvi.02000-18 30728254 PMC6450110

[34] Hu X Tian J Kang H Guo D Liu J Liu D. Transmissible Gastroenteritis Virus Papain-Like Protease 1 Antagonizes Production of Interferon-Beta Through Its Deubiquitinase Activity. BioMed Res Int (2017) 2017:7089091. 10.1155/2017/7089091 29201911 PMC5672592

[35] Channappanavar R Fehr AR Vijay R Mack M Zhao J Meyerholz DK. Dysregulated Type I Interferon and Inflammatory Monocyte-Macrophage Responses Cause Lethal Pneumonia in SARS-CoV-Infected Mice. Cell Host Microbe (2016) 19(2):181–93. 10.1016/j.chom.2016.01.007 PMC4752723 26867177

[36] Comar CE Goldstein SA Li Y Yount B Baric RS Weiss SR. Antagonism of Dsrna-Induced Innate Immune Pathways by NS4a and NS4b Accessory Proteins During MERS Coronavirus Infection. mBio (2019) 10(2):e00319–19. 10.1128/mBio.00319-19 30914508 PMC6437052

[37] Lui PY Wong LY Fung CL Siu KL Yeung ML Yuen KS. Middle East Respiratory Syndrome Coronavirus M Protein Suppresses Type I Interferon Expression Through the Inhibition of TBK1-Dependent Phosphorylation of IRF3. Emerg Microbes Infect (2016) 5:e39. 10.1038/emi.2016.33 27094905 PMC4855074

[38] Yang X Chen X Bian G Tu J Xing Y Wang Y. Proteolytic Processing, Deubiquitinase and Interferon Antagonist Activities of Middle East Respiratory Syndrome Coronavirus Papain-Like Protease. J Gen Virol (2014) 95(Pt 3):614–26. 10.1099/vir.0.059014-0 24362959

[39] Felgenhauer U Schoen A Gad HH Hartmann R Schaubmar AR Failing K. Inhibition of SARS-CoV-2 by Type I and Type III Interferons. J Biol Chem (2020) 295(41):13958–64. 10.1074/jbc.AC120.013788 PMC7549028 32587093

[40] Vanderheiden A Ralfs P Chirkova T Upadhyay AA Zimmerman MG Bedoya S. Type I and Type III Interferons Restrict SARS-CoV-2 Infection of Human Airway Epithelial Cultures. J Virol (2020) 94(19):e00985–20. 10.1128/jvi.00985-20 32699094 PMC7495371

[41] Lei X Dong X Ma R Wang W Xiao X Tian Z. Activation and Evasion of Type I Interferon Responses by SARS-Cov-2. Nat Commun (2020) 11(1):3810. 10.1038/s41467-020-17665-9 32733001 PMC7392898

[42] Kint J Fernandez-Gutierrez M Maier HJ Britton P Langereis MA Koumans J. Activation of the Chicken Type I Interferon Response by Infectious Bronchitis Coronavirus. J Virol (2015) 89(2):1156–67. 10.1128/JVI.02671-14 PMC4300645 25378498

[43] Luo J Fang L Dong N Fang P Ding Z Wang D. Porcine Deltacoronavirus (PdcoV) Infection Suppresses RIG-I-Mediated Interferon-β Production. Virology (2016) 495:10–7. 10.1016/j.virol.2016.04.025 PMC7111668 27152478

[44] Zhu X Fang L Wang D Yang Y Chen J Ye X. Porcine Deltacoronavirus Nsp5 Inhibits Interferon-Beta Production Through the Cleavage of NEMO. Virology (2017) 502:33–8. 10.1016/j.virol.2016.12.005 PMC7111669 27984784

[45] Zhu X Wang D Zhou J Pan T Chen J Yang Y. Porcine Deltacoronavirus Nsp5 Antagonizes Type I Interferon Signaling by Cleaving Stat2. J Virol (2017) 91:e00003–17. 10.1128/jvi.00003-17 28250121 PMC5411617

[46] Fang P Fang L Ren J Hong Y Liu X Zhao Y. Porcine Deltacoronavirus Accessory Protein NS6 Antagonizes IFN-β Production by Interfering With the Binding of RIG-I/MDA5 to Double-Stranded RNA. J Virol (2018) 92:e00712–18. 10.1128/jvi.00712-18 29769346 PMC6052322

[47] Liu X Fang P Fang L Hong Y Zhu X Wang D. Porcine Deltacoronavirus nsp15 Antagonizes Interferon-β Production Independently of its Endoribonuclease Activity. Mol Immunol (2019) 114:100–7. 10.1016/j.molimm.2019.07.003 PMC7112593 31351410

[48] Szelazek B Kabala W Kus K Zdzalik M Twarda-Clapa A Golik P. Structural Characterization of Human Coronavirus Nl63 N Protein. J Virol (2017) 91(11):e02503–16. 10.1128/jvi.02503-16 28331093 PMC5432860

[49] Wu J Shi Y Pan X Wu S Hou R Zhang Y. SARS-Cov-2 ORF9b Inhibits RIG-I-MAVS Antiviral Signaling by Interrupting K63-Linked Ubiquitination of NEMO. Cell Rep (2021) 34(7):108761. 10.1016/j.celrep.2021.108761 33567255 PMC7857071

[50] Wu J Chen ZJ. Innate Immune Sensing and Signaling of Cytosolic Nucleic Acids. Annu Rev Immunol (2014) 32:461–88. 10.1146/annurev-immunol-032713-120156 24655297

[51] Rehwinkel J Gack MU. RIG-I-Like Receptors: Their Regulation and Roles in RNA Sensing. Nat Rev Immunol (2020) 20(9):537–51. 10.1038/s41577-020-0288-3 PMC7094958 32203325

[52] Gack MU Shin YC Joo CH Urano T Liang C Sun L. TRIM25 RING-Finger E3 Ubiquitin Ligase Is Essential for RIG-I-Mediated Antiviral Activity. Nature (2007) 446(7138):916–20. 10.1038/nature05732 17392790

[53] Okamoto M Kouwaki T Fukushima Y Oshiumi H. Regulation of RIG-I Activation by K63-Linked Polyubiquitination. Front Immunol (2017) 8:1942. 10.3389/fimmu.2017.01942 29354136 PMC5760545

[54] Davis ME Gack MU. Ubiquitination in the Antiviral Immune Response. Virology (2015) 479-480:52–65. 10.1016/j.virol.2015.02.033 25753787 PMC4774549

[55] Arimoto K Takahashi H Hishiki T Konishi H Fujita T Shimotohno K. Negative Regulation of the RIG-I Signaling by the Ubiquitin Ligase RNF125. Proc Natl Acad Sci USA (2007) 104(18):7500–5. 10.1073/pnas.0611551104 PMC1863485 17460044

[56] Zhao C Jia M Song H Yu Z Wang W Li Q. The E3 Ubiquitin Ligase TRIM40 Attenuates Antiviral Immune Responses by Targeting MDA5 and RIG-I. Cell Rep (2017) 21(6):1613–23. 10.1016/j.celrep.2017.10.020 29117565

[57] Liu J Qian C Cao X. Post-Translational Modification Control of Innate Immunity. Immunity (2016) 45(1):15–30. 10.1016/j.immuni.2016.06.020 27438764

[58] Zhou Z Sun Y Yan X Tang X Li Q Tan Y. Swine Acute Diarrhea Syndrome Coronavirus (SADS-CoV) Antagonizes Interferon-β Production Via Blocking IPS-1 and RIG-I. Virus Res (2020) 278(11):197843. 10.1016/j.virusres.2019.197843 31884203 PMC7114844

[59] Xia H Cao Z Xie X Zhang X Chen JY Wang H. Evasion of Type I Interferon by SARS-Cov-2. Cell Rep (2020) 33(1):108234. 10.1016/j.celrep.2020.108234 32979938 PMC7501843

[60] Amor S Fernández Blanco L Baker D. Innate Immunity During SARS-CoV-2: Evasion Strategies and Activation Trigger Hypoxia and Vascular Damage. Clin Exp Immunol (2020) 202(2):193–209. 10.1111/cei.13523 32978971 PMC7537271

[61] Lau SK Lau CC Chan KH Li CP Chen H Jin DY. Delayed Induction of Proinflammatory Cytokines and Suppression of Innate Antiviral Response by the Novel Middle East Respiratory Syndrome Coronavirus: Implications for Pathogenesis and Treatment. J Gen Virol (2013) 94(Pt 12):2679–90. 10.1099/vir.0.055533-0 24077366

[62] Frieman M Heise M Baric R. SARS Coronavirus and Innate Immunity. Virus Res (2008) 133(1):101–12. 10.1016/j.virusres.2007.03.015 PMC2292640 17451827

[63] Spiegel M Pichlmair A Martínez-Sobrido L Cros J García-Sastre A Haller O. Inhibition of Beta Interferon Induction by Severe Acute Respiratory Syndrome Coronavirus Suggests a Two-Step Model for Activation of Interferon Regulatory Factor 3. J Virol (2005) 79(4):2079–86. 10.1128/jvi.79.4.2079-2086.2005 PMC546554 15681410

[64] Huang Q Yu L Petros AM Gunasekera A Liu Z Xu N. Structure of the N-Terminal RNA-Binding Domain of the SARS Cov Nucleocapsid Protein. Biochemistry (2004) 43(20):6059–63. 10.1021/bi036155b 15147189

[65] Chen CY Chang CK Chang YW Sue SC Bai HI Riang L. Structure of the SARS Coronavirus Nucleocapsid Protein RNA-Binding Dimerization Domain Suggests a Mechanism for Helical Packaging of Viral RNA. J Mol Biol (2007) 368(4):1075–86. 10.1016/j.jmb.2007.02.069 PMC7094638 17379242

[66] Dinesh DC Chalupska D Silhan J Koutna E Nencka R Veverka V. Structural Basis of RNA Recognition by the SARS-CoV-2 Nucleocapsid Phosphoprotein. PLoS Pathog (2020) 16(12):e1009100. 10.1371/journal.ppat.1009100 33264373 PMC7735635

[67] Zinzula L Basquin J Bohn S Beck F Klumpe S Pfeifer G. High-Resolution Structure and Biophysical Characterization of the Nucleocapsid Phosphoprotein Dimerization Domain From the Covid-19 Severe Acute Respiratory Syndrome Coronavirus 2. Biochem Biophys Res Commun (2020) 538:54–62. 10.1016/j.bbrc.2020.09.131 33039147 PMC7532810

[68] Yu IM Oldham ML Zhang J Chen J. Crystal Structure of the Severe Acute Respiratory Syndrome (SARS) Coronavirus Nucleocapsid Protein Dimerization Domain Reveals Evolutionary Linkage Between Corona- and Arteriviridae. J Biol Chem (2006) 281(25):17134–9. 10.1074/jbc.M602107200 PMC7946579 16627473

[69] Chang CY Liu HM Chang MF Chang SC. Middle East Respiratory Syndrome Coronavirus Nucleocapsid Protein Suppresses Type I and Type III Interferon Induction by Targeting RIG-I Signaling. J Virol (2020) 94(13):e00099–20. 10.1128/jvi.00099-20 32295922 PMC7307178

[70] Chen K Xiao F Hu D Ge W Tian M Wang W. SARS-Cov-2 Nucleocapsid Protein Interacts With RIG-I and Represses RIG-Mediated IFN-β Production. Viruses (2020) 13(1):47. 10.3390/v13010047 PMC7823417 33396605

[71] Zhou Z Sun Y Xu J Tang X Zhou L Li Q. Swine Acute Diarrhea Syndrome Coronavirus Nucleocapsid Protein Antagonizes Interferon-β Production Via Blocking the Interaction Between TRAF3 and TBK1. Front Immunol (2021) 12:573078. 10.3389/fimmu.2021.573078 33692778 PMC7937869

[72] Tan X Sun L Chen J Chen ZJ. Detection of Microbial Infections Through Innate Immune Sensing of Nucleic Acids. Annu Rev Microbiol (2018) 72:447–78. 10.1146/annurev-micro-102215-095605 30200854

[73] Nakhaei P Genin P Civas A Hiscott J. RIG-I-Like Receptors: Sensing and Responding to RNA Virus Infection. Semin Immunol (2009) 21(4):215–22. 10.1016/j.smim.2009.05.001 19539500

[74] Wies E Wang MK Maharaj NP Chen K Zhou S Finberg RW. Dephosphorylation of the RNA Sensors RIG-I and MDA5 by the Phosphatase PP1 Is Essential for Innate Immune Signaling. Immunity (2013) 38(3):437–49. 10.1016/j.immuni.2012.11.018 PMC3616631 23499489

[75] Kok KH Lui PY Ng MH Siu KL Au SW Jin DY. The Double-Stranded RNA-Binding Protein PACT Functions as a Cellular Activator of RIG-I to Facilitate Innate Antiviral Response. Cell Host Microbe (2011) 9(4):299–309. 10.1016/j.chom.2011.03.007 21501829

[76] Oshiumi H Miyashita M Matsumoto M Seya T. A Distinct Role of Riplet-Mediated K63-Linked Polyubiquitination of the RIG-I Repressor Domain in Human Antiviral Innate Immune Responses. PLoS Pathog (2013) 9(8):e1003533. 10.1371/journal.ppat.1003533 23950712 PMC3738492

[77] Wang W Jiang M Liu S Zhang S Liu W Ma Y. RNF122 Suppresses Antiviral Type I Interferon Production by Targeting RIG-I Cards to Mediate RIG-I Degradation. Proc Natl Acad Sci USA (2016) 113(34):9581–6. 10.1073/pnas.1604277113 PMC5003265 27506794

[78] Bowie AG Unterholzner L. Viral Evasion and Subversion of Pattern-Recognition Receptor Signalling. Nat Rev Immunol (2008) 8(12):911–22. 10.1038/nri2436 PMC7097711 18989317

[79] Chiang JJ Davis ME Gack MU. Regulation of RIG-I-Like Receptor Signaling by Host and Viral Proteins. Cytokine Growth Factor Rev (2014) 25(5):491–505. 10.1016/j.cytogfr.2014.06.005 25023063 PMC7108356

[80] Chiang C Pauli EK Biryukov J Feister KF Meng M White EA. The Human Papillomavirus E6 Oncoprotein Targets USP15 and TRIM25 to Suppress RIG-I-Mediated Innate Immune Signaling. J Virol (2018) 92(6):e01737–17. 10.1128/jvi.01737-17 29263274 PMC5827370

[81] Sanchez-Aparicio MT Feinman LJ Garcia-Sastre A Shaw ML. Paramyxovirus V Proteins Interact With the RIG-I/TRIM25 Regulatory Complex and Inhibit RIG-I Signaling. J Virol (2018) 92(6):e01960–17. 10.1128/jvi.01960-17 29321315 PMC5827389

[82] He X Zhu Y Zhang Y Geng Y Gong J Geng J. RNF34 Functions in Immunity and Selective Mitophagy by Targeting MAVS for Autophagic Degradation. EMBO J (2019) 38(14):e100978. 10.15252/embj.2018100978 31304625 PMC6627233

[83] Wu Y Jin S Liu Q Zhang Y Ma L Zhao Z. Selective Autophagy Controls the Stability of Transcription Factor IRF3 to Balance Type I Interferon Production and Immune Suppression. Autophagy (2020) 1–14. 10.1080/15548627.2020.1761653 PMC8205069 32476569

