# Supplementary figures and images for: A Comparative Analysis of Coronavirus Nucleocapsid (N) Proteins Reveals the SADS-CoV N Protein Antagonizes IFN-β Production by Inducing Ubiquitination of RIG-I
Source: Front Immunol. 2021 Jun 16;12:688758. doi: 10.3389/fimmu.2021.688758 (PMC8242249; doi:10.3389/fimmu.2021.688758)

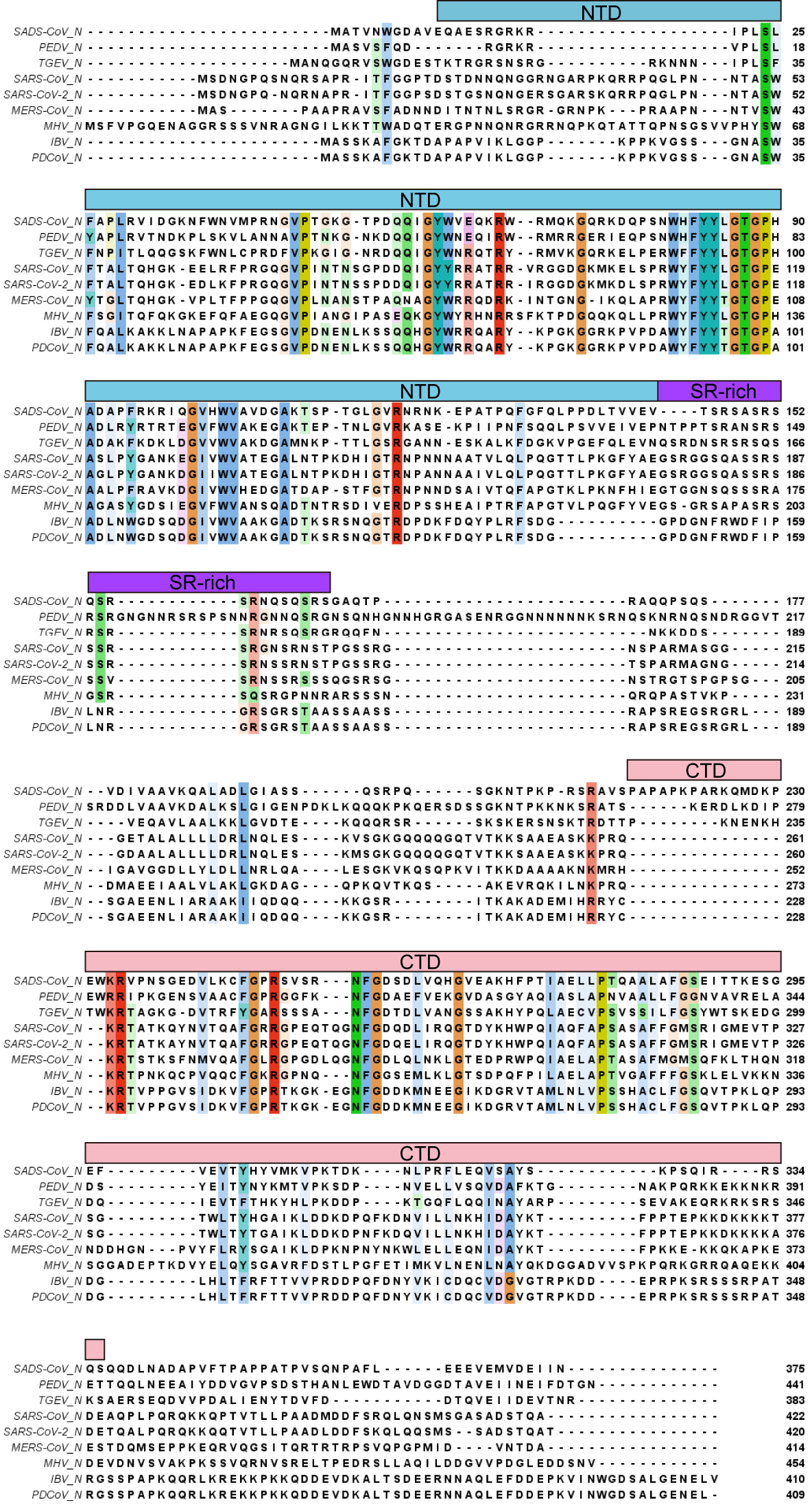

Supplement: Supplementary Figure S1 — Multiple amino acid sequence alignment of the N proteins among the representative CoVs shown in Figure 1B. The alignment was conducted by ClustalW and the image was modified by Jalview 2.2. The default color scheme was used (http://www.jalview.org/help/html/colourSchemes/clustal.html) and 50% conservation was set to be the threshold for color visibility. The sequences corresponding to the NTD, CTD and RS-rich region of the SADS-CoV N were marked above. [file Image_1.tif]
